# *In vitro* antifungal susceptibility pattern of *Candida* species isolated from gastroesophageal candidiasis 

**Published:** 2021

**Authors:** Mohsen Mirshekar, Mohammad Hassan Emami, Rasoul Mohammadi

**Affiliations:** 1 *Department of Medical Parasitology and Mycology, School of Medicine, Isfahan University of Medical Sciences, Isfahan, Iran*; 2 *Poursina Hakim Digestive Diseases Research Center, Isfahan University of Medical Sciences, Isfahan, Iran*; 3 *Infectious Diseases and Tropical Medicine Research Center, Isfahan University of Medical Sciences, Isfahan, Iran *

**Keywords:** antifungal susceptibility testing, Candida spp., gastroesophageal

## Abstract

**Aim:**

The current study aimed to determine the antifungal susceptibility profile of *Candida* species isolated from gastroesophageal lesions

**Background:**

Gastroesophageal candidiasis is a common infection among HIV/AIDS patients and those who are taking PPI and H2RAs drugs*. *More than 20 *Candida* spp. can cause different types of mucocutaneous infections in humans. The present study was conducted to assess the antifungal susceptibility testing of clinical *Candida* spp. isolated from gastroesophageal lesions.

**Methods:**

Forty-eight clinical samples were collected from 60 patients undergoing endoscopy. All isolates were identified by molecular techniques (PCR-RFLP). The profiles of the susceptibility of *Candida* spp. to seven antifungal agents, i.e. amphotericin B, fluconazole, itraconazole, luliconazole, voriconazole, posaconazole, and caspofungin, were evaluated using broth microdilution.

**Results:**

The susceptibility profile of *Candida* isolates revealed 100% sensitivity to amphotericin B, caspofungin, and voriconazole. Moreover, fluconazole- (6.5%) and itraconazole-resistant (2.1%) isolates were observed.

**Conclusion:**

With regard to the increase in fluconazole-resistant *Candida* species, it is necessary to determine the in vitro antifungal susceptibility pattern of clinical isolates for the best management of infection and to prevent the emergence of drug resistant isolates.

## Introduction

*Candida* species, as normal flora, can colonize the surface epithelium of the alimentary tract of healthy individuals. They are the main cause of mucocutaneous fungal infections normally categorized as oropharyngeal, esophageal, gastritis, and vulvovaginal candidiasis (1). Gastroesophageal candidiasis is a prevalent infection among HIV patients and individuals who use chemotherapy, corticosteroid therapy, antibiotic therapy, or radiotherapy (2). Diabetes mellitus, gastric surgery, acid suppression, and esophageal motility disorders are other risk factors for gastroesophageal candidiasis (3). More than 20 *Candida* spp. can cause different types of mucocutaneous infections in humans, and the most frequent pathogens are *C. albicans*, *C. glabrata, C. parapsolosis, C. krusei, C. dubliniensis, *and *C. tropicalis *(4). The pathogenicity of these yeasts changes from species to species and depends on the degree of impairment of the immune system. Antifungal resistance is a significant concern in clinical practice and is increasingly documented (5, 6). Increased use of antifungal drugs among immunosuppressed patients has been considered as a main reason for the emergence of drug resistant *Candida* species and impotent responses to antifungal agents (7). Non-selectively treatment of gastroesophageal candidiasis without *in vitro* antifungal susceptibility testing can lead to the elimination of drug sensitive *Candida* species by more intrinsically resistant species such as *Candida glabrata* and *Candida krusei*. The present study was conducted to assess the antifungal susceptibility testing of clinical *Candida* spp. isolates from gastroesophageal lesions. 

## Methods

The investigation protocol was reviewed and approved by the Ethics Committee of Isfahan University of Medical Sciences (IR.MUI.MED.REC.1398.341). Sixty clinical samples were obtained from patients undergoing endoscopy in Poursina Hakim Digestive Diseases Research Center, Isfahan University of Medical Sciences, Isfahan, Iran. Inclusion criteria were probable gastritis and esophagitis in accordance with clinical signs and no history of antifungal consumption within 14 days prior to the physician visit. The age range of patients was 21 to 83 years, and the male to female ratio was 25/35. Clinical specimens were obtained from gastric tissue biopsy (n=22), gastric juice (n=21), and esophageal biopsy specimens (n=17). All *Candida* species used in this study (48 isolates) were previously identified to the species level by the PCR-RFLP method (8). Briefly, the ITS1-5.8SrDNA-ITS2 region was amplified using ITS1 and ITS4 primers, and species identification was performed through specific electrophoretic profiles of isolates after digestion with the restriction enzyme *Msp*I. 

**Antifungal susceptibility testing (AFST)**

Minimum inhibitory concentration (MICs) was assessed by the Clinical and Laboratory Standards Institute (CLSI) M27-A3 and M27-S4 documents (9, 10). Fluconazole (Pfizer Central Research, Sandwich, United Kingdom), itraconazole (Janssen Research Foundation, Beerse, Belgium), amphotericin B (Bristol-Myers-Squib, Woerden, and the Netherlands), caspofungin (Merck Sharp & Dohme, Haarlem, The Netherlands), posaconazole (Schering-Plough, Kenil-worth, USA), voriconazole (Pfizer Central Research, UK), and luliconazole (Sigma Chemical Co., St. Louis, MO, USA) were applied for preparation of the CLSI microdilution trays. Antifungal agents were diluted in the RPMI-1640 medium (Sigma Chemical Co., St. Louis, MO, USA) buffered to pH 7.0 with 0.165 M morpholinepropanesulfonic acid (MOPS) (Sigma Chemical Co., St. Louis, MO, USA) with L-glutamine and without bicarbonate to yield two-times concentrations and were dispensed into 96-well microdilution trays at a final concentration of 0.064–64 μg/ml for fluconazole, 0.016–8 μg/ml for caspofungin, 0.016–16 μg/ml for itraconazole, voriconazole, amphotericin B, posaconazole, and 0.008–16 μg/ml for luliconazole. Plates were stored at -70 °C until use. All identified *Candida *spp*. *were cultured on malt extract agar (MEA, Difco) and incubated at 35 °C. The inoculum volumes were prepared by harvesting the cell from 24 h old cultures and were adjusted spectrophotometrically in normal saline to optical densities ranging 75%-77% transmission. Final inoculum sizes ranged from 2.5×10^3^ to 5×10^3^ CFU/ml. MIC results for all agents were determined visually following 24 h of incubation at 35 °C, as the lowest concentration of drug that caused complete (such as amphotericin B) or significant (>50%) growth decrease levels (such as fluconazole) (9).* Candida krusei *(ATCC 6258) and* C. parapsilosis *(ATCC 22019) strains were applied as quality controls. Table 1 shows the interpretive guidelines for *in vitro* susceptibility testing of *Candida* species according to M27-S4 and M60 documents (10-12).

**Statistical Analysis**

The MIC range, MIC_50_ (the minimum concentrations of antifungals that were able to inhibit 50% of growth), and MIC_90_ (the minimum concentrations of drugs that were able to inhibit 90% of growth) were determined. Data was analyzed using SPSS software version 23 (Chicago, IL, USA). Correlations between antifungal susceptibility rates and species distribution were adjusted using Fisher's exact test and Mann-Whitney *U-*test. A *p*-value of < 0.05 was considered significant.

**Table 1:** Interpretive guidelines for *in vitro* susceptibility testing of *Candida* species

Antifungal Agent	*Candida* Species	MIC Breakpoints (µg/mL)			
		S	I	SDD	R
Fluconazole	*C. albicans*	≤ 2	-	4	≥ 8
	*C. glabrata*	-	-	≤32	≥ 64
	*C. parapsilosis*	≤ 2	-	4	≥ 8
Voriconazole	*C. albicans*	≤ 0.12	0.25-0.5	-	≥ 1
	*C. glabrata * ^a^	-	-	-	-
	*C. parapsilosis*	≤ 0.12	0.25-0.5	-	≥ 1
Itraconazole	*C. albicans*	≤ 0.125	-	0.25-0.5	≥ 1
	*C. glabrata*	≤ 0.125	-	0.25-0.5	≥ 1
	*C. parapsilosis*	≤ 0.125	-	0.25-0.5	≥ 1
Posaconazole ^b^	*C. albicans*	-	-	-	-
	*C. glabrata*	-	-	-	-
	*C. parapsilosis*	-	-	-	-
Luliconazole ^c^	*C. albicans*	-	-	-	-
	*C. glabrata*	-	-	-	-
	*C. parapsilosis*	-	-	-	-
Caspogungin	*C. albicans*	≤ 0.25	0.5	-	≥ 1
	*C. glabrata*	≤ 0.12	0.25	-	≥ 0.5
	*C. parapsilosis*	≤ 2	4	-	≥ 8
Amphotericin B ^b^	*C. albicans*	-	-	-	2
	*C. glabrata*	-	-	-	2
	*C. parapsilosis*	-	-	-	2

S: susceptible; I: intermediate; SDD: susceptible dose dependent; R: resistant;^a^ For *C. glabrata* and voriconazole current data is insufficient to demonstrate a correlation between *in vitro* susceptibility testing and clinical outcome. ^b ^For posaconazole and amphotericin B, epidemiological cutoff values (ECV) have been replaced for *Candida* spp. with no breakpoints. ^c ^For luliconazole, there is no breakpoint or EVC for *Candida* spp.

## Results

Forty-eight out of 60 specimens grew on Sabouraud dextrose agar (SDA) (Biolife, Italy) with chloramphenicol (Merck, Germany). Thirty-six isolates were *C. albicans *(75%), followed by *C. glabrata* (n=8, 16.6%), *C. parapsilosis* (n=2, 4.2%), and *C. famata* (n =2, 4.2%). The main clinical symptoms of gastroesophageal candidiasis among patients were gastritis (34%), dysphagia (26.4%), acid reflux (19.8%), and flatulence (19.8%). The most prevalent risk factors were antibiotic consumption (8.8%), diabetes mellitus (6.6%), and gastric bypass surgery (2.2%). The MIC ranges of antifungal agents were as follows: *C. albicans*: (AMB, 0.016–1 μg/mL), (CAS, 0.016–0.032 μg/mL), (VOR, 0.016–0.064 μg/mL), (FLC, 0.064–16 μg/mL), (POS, 0.016–1 μg/mL), (ITR, 0.016–0.05 μg/mL), (LLCZ, 0.008–2 μg/mL); *C. glabrata*: (AMB, 0.016–1 μg/mL), (CAS, 0.016–0.125 μg/mL), (VOR, 0.016–0.25 μg/mL), (FLC, 4–32 μg/mL ), (POS, 0.125–1 μg/mL), (ITR, 0.032–0.5 μg/mL), (LLCZ, 0.008–4 μg/mL); *C. parapsilosis*: (AMB, 0.016–0.5 μg/mL ), (CAS, 0.016 μg/mL), (VOR, 0.016–0.5 μg/mL), (FLC, 0.125–8 μg/mL ), (POS, 0.016–1 μg/mL), (ITR, 0.016–1 μg/mL), (LLCZ, 1 μg/mL); and *C. famata*: (AMB, 0.016–0.25 μg/mL), (CAS, 0.016–0.064 μg/mL), (VOR, 0.016–0.064 μg/mL), (FLC, 0.125–0.5 μg/mL), (POS, 0.016–0.5 μg/mL), (ITR, 0.016–1 μg/mL), (LLCZ, 0.008–1 μg/mL). Tables 2-4 show the MIC of antifungal agents in detail, MIC_50_, MIC_90_, geometric mean (GM), and *in vitro* susceptibility patterns of *Candida* species, respectively. 

**Table 2:** The MIC of antifungal agents among *Candida* spp. isolated from gastroesophageal lesions

Antifungal Agents	Minimum Inhibitory Concentration (μg/mL)											
	≤0.016	0.032	0.064	0.128	0.256	0.512	1	2	4	8	16	≥32
Amphotericin B	15	2	0	9	13	7	2	0	0	0	0	0
Caspofungin	38	3	6	1	0	0	0	0	0	0	0	0
Voriconazole	23	10	11	1	2	1	0	0	0	0	0	0
Fluconazole	0	0	1	5	7	2	2	7	16	2	3	3
Posaconazole	5	3	7	9	8	9	7	0	0	0	0	0
Itraconazole	11	6	12	9	3	5	2	0	0	0	0	0
Luliconazole	14	2	4	3	4	11	5	4	1	0	0	0

**Data Analysis**

Fisher’s exact test showed that the association between MIC and *Candida* species was not statistically significant (*p* = 0.74). The MIC_50_ and MIC_90_ values were considered as the minimum concentrations of antifungal agents being able to inhibit 50% and 90% of the growth of clinical *Candida* strains, respectively.

## Discussion

Little is known about the antifungal susceptibility profile of *Candida* species isolated from gastroesophageal candidiasis. In this study, 48 clinical *Candida* species were obtained from 60 patients who took proton pump inhibitors (PPI) (omeprazole, pantoprazole, rabeprazole, lansoprazole, and nolpaza) or histamine receptor antagonist (H2RAs) (ranitidine) drugs. The culture positivity rate obtained in the present investigation (80%) was in accordance with the study reported by Mulu et al. (13) and was relatively higher than the culture positivity rate (69%) reported in a study performed in Ethiopia (7). Esophageal and gastric candidiasis routinely responds well to antifungal agents. In comparison to oropharyngeal candidiasis, the treatment of gastroesophageal candidiasis is commonly systemic rather than topical (14). The long-term use of various antifungals in clinics has resulted in the increase of multidrug resistance (MDR) isolates such as *C. glabrata*, which is a significant issue facing healthcare worldwide and causes a major impediment to antifungal therapy (15). The haploid nature of the microorganism may develop MDR in *C. glabrata. *This fungus can express resistant mutations under protracted drug exposure (16). None of the *C. glabrata* in the present research were MDR, which may be due to a lack of long-term exposure to antifungal drugs or the appropriate dose of the antifungals being prescribed by physicians in this area. The most frequently used antifungal agents for the treatment of gastroesophageal candidiasis is oral fluconazole 200–400 mg/day for 2–3 weeks, (OR) 400 mg of fluconazole intravenously daily, as the alternative therapy for patients who may not be able to tolerate oral fluconazole (17). Another options are voriconazole 200 mg twice/day for 2–3 weeks and itraconazole 200 mg daily. Medical treatment with posaconazole 400 mg orally twice daily is reported to be remarkably effective. Polyenes such as amphotericin B deoxycholate (ABD) (0.3–0.7 mg/kg/day) can also be useful; however, they have substantial side effects, such as severe nephrotoxicity, and thus, clinicians must avoid their routine use (18). Echinocandins (anidulafungin, caspofungin, and micafungin) are appropriate drugs to replace with polyenes for patients with renal failure. In the present investigation, 100% of the *Candida* isolates were susceptible to CAS, with an MIC range of 0.016–0.125 µg/mL. These findings are in agreement with those of the national SENTRY antifungal surveillance and Global ARTEMIS studies (19, 20). The present study determined the MIC of the abovementioned drugs as well as luliconazole (also known as NND-502), a newly imidazole antifungal agent. The MIC of luliconazole against *Candida* spp. has been revealed to be higher than that against molds such as dermatophytes; however, it has a better effect than those of terbinafine, amorolfine, and bifonazole (21, 22). The current results showed that 10.4% of the *Candida* isolates had a MIC range above 1 µg/mL for luliconazole. Luliconazole has been shown to have MIC similar to that of 5-fluorocytosine (5-FC) against *Candida* spp., but one of the major limitations of the current study was the lack of 5-FC among the antifungal drugs tested on clinical isolates. The susceptibility profile of *Candida* isolates revealed 100% sensitivity to amphotericin B, caspofungin, and voriconazole, but fluconazole- (6.5%) and itraconazole-resistant (2.1%) strains were isolated. Fluconazole, a triazole antifungal agent, is efficient against *Candida *species as well as other fungal infections in immunocompromised patients (23). Additionally, triazoles are prescribed as first-line therapy for esophageal and gastric candidiasis among hospitalized patients (24); nevertheless, treatment with azole drugs for gastroesophageal candidiasis sometimes causes considerable side effects including nausea, vomiting, abdominal pain, and diarrhea (17). Many investigations have shown that the sensitivity of *C. tropicalis, C. glabrata*, and *C. krusei *to amphotericin B and fluconazole has been reduced during two past decades (25-27). In accordance with this data, the incidence of fluconazole-resistant *Candida* isolates in the present investigation was 6.5%. The molecular mechanisms of resistance to fluconazole could be summarized as follows: upregulation of the target enzyme, alteration of target site, expansion of bypass pathways, and drug concentration decrement (28). Shokohi et al. (29) detected the MICs of antifungal agents for *Candida* species isolated from oropharyngeal lesions of cancer patients. In their study, 4.4%, 7.2%, 2.9%, and 2.9% of clinical isolates were resistant to amphotericin B, itraconazole, caspofungin, and fluconazole, respectively, whereas the clinical isolates of the present investigation showed 100% sensitivity to amphotericin B and caspofungin. In accordance with the current findings, Badiee et al. (30) revealed that all species were sensitive to amphotencin B and caspofungin. There is a disorder of the esophagus called cardiac achalasia which can cause stasis of secretions and food in the esophagus, which may lead to the overgrowth of *Candida *species and development of esophageal lesions (31). None of the patients enrolled in the present study had cardiac achalasia. 

In conclusion, with regard to the increase of fluconazole-resistant *Candida* species, it is necessary to determine the in vitro antifungal susceptibility pattern of clinical isolates for the best management of infection and to prevent the emergence of drug resistant isolates. Moreover, the current results suggest the use of caspofungin, amphotericin B, and voriconazole as first-line therapies against clinical isolates of *Candida *species in patients with gastroesophageal candidiasis.

**Table 3:** MIC Range, MIC_50_, MIC_90_, and geometric mean (GM) of the seven antifungals

*Candida* Species	MIC Range (μg/mL)	MIC_50_ (μg/mL)	MIC_90_ (μg/mL)	Geometric Mean
*C. albicans*	AMB (0.016–1) CAS (0.016–0.032) VOR (0.016–0.064) FLC (0.064–16) POS (0.016–1) ITR (0.016–0.05) LLCZ (0.008–2)	0.125 0.016 0.016 2 0.125 0.064 0.25	0.5 0.032 0.064 4 0.5 0.125 2	0.107 0.016 0.024 1.3 0.138 0.056 0.126
*C. glabrata*	AMB (0.016–1) CAS (0.016–0.125) VOR (0.016–0.25) FLC (4–32) POS (0.125–1) ITR (0.032–0.5) LLCZ (0.008–4)	0.125 0.064 0.064 16 0.5 0.25 0.016	1 0.125 0.25 32 1 0.5 4	0.115 0.049 0.07 14.67 0.458 0.206 0.082
*C. parapsilosis*	AMB (0.016–0.5) CAS (0.016) VOR (0.016–0.5) FLC (0.125–8) POS (0.016–1) ITR (0.016–1) LLCZ (1)	0.016 0.016 0.016 0.125 0.016 0.016 1	0.5 0.016 0.5 8 1 1 1	0.089 0.016 0.089 1 0.126 0.126 1
*C. famata*	AMB (0.016–0.25) CAS (0.016–0.064) VOR (0.016–0.064) FLC (0.125–0.5) POS (0.016–0.5) ITR (0.016–1) LLCZ (0.008–1)	0.016 0.016 0.016 0.125 0.016 0.016 0.008	0.25 0.064 0.064 0.5 0.5 1 1	0.063 0.032 0.032 0.25 0.089 0.126 0.089

**Table 4:** *In vitro* susceptibility patterns of *Candida* species isolates from gastroesophageal candidiasis

*Candida* spp.	Antifungal Pattern	Antifungal Agents						
		AMB	CAS	VOR ^b^	FLC	POS ^a^	ITR	LLCZ ^a^
*C. albicans*	S I / (SSD) R	36 0 0	36 0 0	36 0 0	20 (14) 2	- - -	33 (3) 0	- - -
*C. glabrata*	S I / (SSD) R	0 8 0	8 0 0	- - -	0 (8) 0	- - -	3 (5) 0	- - -
*C. parapsilosis*	S I / (SSD) R	2 0 0	2 0 0	1 1 0	1 0 1	- - -	1 0 1	- - -
*C. famata* ^c^	S I / (SSD) R	- - -	- - -	- - -	- - -	- - -	- - -	- - -
Total	S I / (SSD) R	38 8 0	46 0 0	37 1 0	21 (22) 3	- - -	37 (8) 1	- - -

S: susceptible; I: intermediate; SDD: susceptible dose dependent; R: resistant; AMB: amphotericin B; CAS: caspofungin; VOR: voriconazole; FLC: fluconazole; POS: posaconazole; ITR: itraconazole; LLCZ: luliconazole. ^a^ Posaconazole and luliconazole have no breakpoint in the new version of CLSI. ^b ^For *C. glabrata* and voriconazole, current data is insufficient to demonstrate a correlation between in vitro susceptibility testing and clinical outcome. ^c ^All antifungals have no breakpoints for uncommon species such as *C. famata*
